# Studies of Thermal Conductivity of Graphite Foil-Based Composite Materials

**DOI:** 10.3390/ma18020233

**Published:** 2025-01-08

**Authors:** Vladimir A. Shulyak, Nikolai S. Morozov, Roman A. Minushkin, Viktor Yu. Gubin, Dmitriy V. Vakhrushin, Alexandra V. Gracheva, Ildar Kh. Nigmatullin, Sergei N. Chebotarev, Viktor V. Avdeev

**Affiliations:** Department of Chemistry, Lomonosov Moscow State University, Moscow 119991, Russia; shulyak.v@unichimtek.ru (V.A.S.); minushkinroman@yandex.ru (R.A.M.); classic_fond@mail.ru (V.Y.G.); vakhrushin_dmitry@mail.ru (D.V.V.); gracheva.a@inumit.ru (A.V.G.); nigmatullin.i@inumit.ru (I.K.N.); chebotarev.s@inumit.ru (S.N.C.); avdeev@highp.chem.msu.ru (V.V.A.)

**Keywords:** thermal conductivity coefficient, flexible graphite foil, natural graphite, artificial graphite, aluminum, copper, heat flux, Fourier’s law, finite element method

## Abstract

We have proposed and developed a method for measuring the thermal conductivity of highly efficient thermal conductors. The measurement method was tested on pure metals with high thermal conductivity coefficients: aluminum (99.999 wt.% Al) and copper (99.990 wt.% Cu). It was demonstrated that their thermal conductivities at a temperature of T = 22 ± 1 °C were <λ_Al_> = 243 ± 3 W/m·K and <λ_Cu_> = 405 ± 4 W/m·K, which was in good agreement with values reported in the literature. Artificial graphite ($\rho_{G1}$ = 1.8 g/cm^3^) and natural graphite ($\rho_{G2}$ = 1.7 g/cm^3^) were used as reference carbon materials; the measured thermal conductivities were <λ_G1_> = 87 ± 1 W/m·K and <λ_G2_> = 145 ± 3 W/m·K, respectively. It is well established that measuring the thermal conductivity coefficient of thin flexible graphite foils is a complex metrological task. We have proposed to manufacture a solid rectangular sample formed by alternating layers of thin graphite foils connected by layers of ultra-thin polyethylene films. Computer modelling showed that, for equal thermal conductivities of solid products made of compacted thermally exfoliated graphite and products made of a composite material consisting of 100 layers of thin graphite foil and 99 layers of polyethylene, the differences in temperature fields did not exceed 1%. The obtained result substantiates our proposed approach to measuring thermal conductivity of flexible graphite foil by creating a multi-layer composite material. The thermal conductivity coefficient of such a composite at room temperature was <λ_GF_> = 184 ± 6 W/m·K, which aligns well with measurements by the laser flash method.

## 1. Introduction

Measuring the thermal conductivity of materials is a key element of modern science and technology, affecting the efficiency and safety of many industrial and household processes [1,2,3]. Modern technologies require materials with high thermal conductivity or, conversely, thermal insulation properties, which are used in various fields of science and technology [4,5,6].

Thermal conductivity of materials directly affects their ability to transfer heat, which is especially important in electronics [7,8,9]. Efficient heat dissipation by radiators and cooling systems depends on materials with high thermal conductivity coefficients [10,11,12]. In power engineering and mechanical engineering, materials with high thermal conductivity are used for efficient heat removal in order to prevent equipment overheating. Measuring the thermal conductivity coefficient allows for a proper selection of materials for specific operating conditions, which increases the efficiency and productivity of heat exchange processes. Materials with low thermal conductivity are used in thermal insulation of buildings and industrial equipment to minimize heat losses [13,14,15]. Accurate measurement of thermal conductivity coefficient allows for the optimization of insulating materials, which leads to a decrease in energy costs for heating and cooling [16,17,18].

Also, this work demonstrates, for the first time, a layered composite material based on graphite foil. Flexible graphite foil (GF) is a functional material with pre-specified properties which are defined by the production process of the material [19,20]. GF is a derivative of natural crystalline graphite; the treatment of the latter with strong oxidizing Brønsted acids yields inclusion compounds, termed graphite intercalation compounds (GIC) [21,22,23]. Quick heating of GIC or oxidized graphite (adduct of GIC and water) can cause the guest compound to create exfoliation pressure, which significantly changes the morphology of the powder, leading to the formation of thermally expanded graphite. Pressing of thermally exfoliated graphite powder yields a mechanically strong compact, termed (flexible) graphite foil [24,25,26].

The physical and mechanical properties of GF depend on many parameters of its production; for example, we have previously demonstrated the dependence of thermal conductivity of the material on its density [20], and linear dependences of both the thermal diffusivity and thermal conductivity on density have also been recorded. The actual value of the thermal conductivity coefficient for GF with a specified density, e.g., 1.0 g/cm^3^, can vary from 170 W/m·K [20] to 210 W/m·K [23,27,28] in different works, which confirms the previously mentioned statement on the relationship between the material’s production conditions and its properties.

The bulk density of GF is usually in the range of 0.7–1.8 g/cm^3^ [29,30]. However, the maximum density of GF can reach 2.26 g/cm^3^, since its crystal lattice is of the same type as in graphite.

The classic materials used as heat conductors are copper (*ρ* = 8.9 g/cm^3^, *λ_Cu_* = 405 W/m·K [31,32]), an affordable material with very high thermal conductivity, and aluminum (*ρ* = 2.7 g/cm^3^, *λ_Cu_* = 243 W/m·K [33,34]), an available and cheap material with fairly high thermal conductivity. Of particular interest for our study is the specific thermal conductivity, which was determined according to the following equation (Equation (1)):

(1) $$X=\frac{\lambda }{\rho }\cdot 1000$$

where X is the specific thermal conductivity of a material [(W·m^2^)/(kg·K)]; λ is the thermal conductivity of the material; and ρ is the material density.

Using the formula, the above-mentioned values can be transformed into the following X values: 45 (W·m^2^)/(kg·K) for copper; 90 (W·m^2^)/(kg·K) for aluminum; 184 (W·m^2^)/(kg·K) for graphite foil with a density of 1.0 g/cm^3^; 186 (W·m^2^)/(kg·K) for graphite foil with a density of 1.8 g/cm^3^; and 897 (W·m^2^)/(kg·K) for quasi-monocrystalline graphite with a density of 2.2 g/cm^3^ [35,36]. The specific thermal conductivity of graphite foil is significantly higher than that of copper and aluminum, which makes it attractive for applications in those branches of science and technology where the product weight is of significant importance (microelectronics, aircraft engineering, batteries, etc.) [37,38]. Comparison of the thermal conductivity coefficients for GF and graphite quasi-single crystals shows that there is significant potential for increasing the thermal conductivity of GF.

Another allotropic modification of carbon, graphene, has a high thermal conductivity coefficient (2000 W/m·K). The laser flash method, a common method for measuring thermal conductivity, is ambiguous due to the predetermined value of the heat capacity of the material [39,40]. Therefore, a more reliable method for measuring thermal conductivity is needed.

In order to develop new approaches to obtaining GF, a device is needed that would be capable of measuring the thermal conductivity of materials obtained with high accuracy. In our previous work [41], we demonstrated the first version of the developed based on Fourier’s law. This device was successfully calibrated and showed excellent results with high-purity aluminum samples. The aim of this work is to expand the range of measurable thermal conductivities of materials, as well as to measure the thermal conductivity coefficient of a composite graphite foil-based material.

## 2. Materials and Methods

### 2.1. Base Materials with High Thermal Conductivity

**Figure 1:**
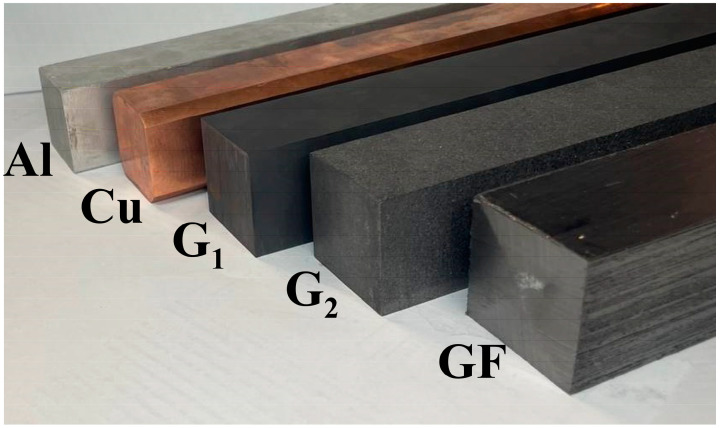
Samples for measuring thermal conductivity coefficient.

Copper and aluminum (Figure 1) are metals with among the highest thermal conductivities of all metals, making them efficient heat conductors.

The efficiency of such materials is especially important in heating systems, where copper/aluminum pipes allow for quick transfer of heat from boiler to radiators, ensuring a comfortable room temperature. Likewise, in cooling systems, copper/aluminum elements efficiently remove heat, thus reducing the usage of compressors and increasing their service life.

In electronics, copper/aluminum materials are used as heat sinks and radiators that can quickly remove heat from critical components, preventing them from overheating. This is especially important under conditions of high load on electronics, which allows for an increase in its durability and efficiency.

**Figure 2:**
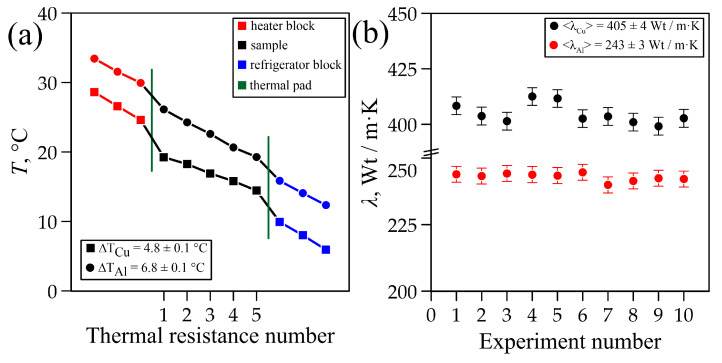
Distribution of the temperature (**a**) and thermal conductivity (**b**).

Studies of an aluminum sample (99.999 wt.% Al) in an experimental setup are detailed in our previous work (Figure 2) [41]. In the present work, in addition to the aluminum sample, we also studied samples of pure copper (99.990 wt.% Cu) and of a composite graphite foil-based material (more details thereon will be given in Section 2.2). The geometric dimensions of the samples were 40 × 40 × 300 mm (Figure 1).

In a series of 10 measurements with a temperature difference of ∆T = 4.8 ± 0.1 °C (Figure 2a), the thermal conductivity of the copper sample was <λ> = 405 ± 4 W/m·K (Figure 2b). According to [42], the thermal conductivity of copper ranges between 327 and 418 W/m·K, but the recommended value is considered to be 400 W/m·K.

### 2.2. Structural and Functional Materials

**Figure 3:**
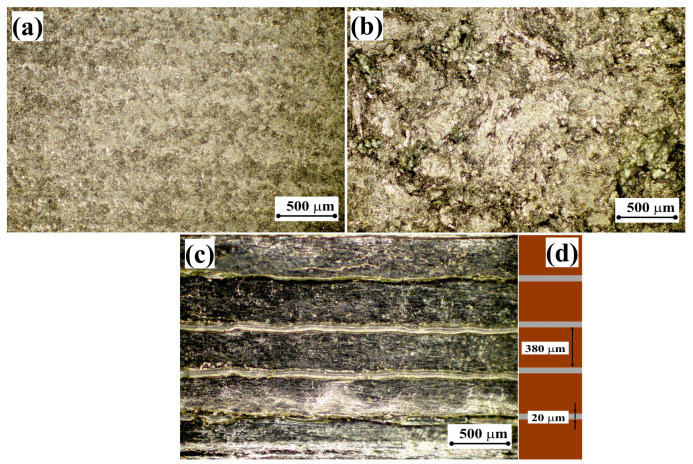
Sample of graphite foil-based composite material. Artificial graphite sample G_1_ (**a**), natural graphite sample G_2_ (**b**), graphite foil-based composite material (**c**), geometric interpretation of the GF sample for modeling purposes (**d**).

Successful measurements of the thermal conductivities of aluminum and copper samples confirm that our experimental setup can be used for measuring the thermal conductivity coefficients of carbon materials, namely, a sample of artificial graphite G_1_ ($\rho_{G1}$ = 1.8 g/cm^3^), a sample of natural graphite G_2_ ($\rho_{G2}$ = 1.7 g/cm^3^), and a unique composite anisotropic graphite foil-based material (Figure 1). Microstructures of the studied samples are shown in Figure 3a–c.

The graphite foil-based composite sample for thermal conductivity measurements was obtained in several stages. First, natural graphite powder was mixed with fuming nitric acid at a graphite/acid ratio of 1:0.8, and the mixture was stirred manually for 1 h. After that, distilled water was added at a graphite/water ratio of 1:30, hydrolysis was carried out for 5 min, and excess acid was removed using a porous filter. The obtained material was dried in a convection cabinet for 5 h at a temperature of 50 °C. Thermal exfoliation was conducted at a temperature of 1000 °C in air; the exposure duration did not exceed 2 s. The obtained powder was rolled to form a flexible graphite foil with a thickness of 0.38 mm and a density of 1.0 g/cm^3^. After that, 40 × 300 mm strips were cut out of this graphite foil.

The test sample was obtained by successively alternating 20 μm thick graphite foil and polyethylene layers. The two materials were stacked so that there were 102 foil layers (the so-called “in-plane” technology). This stack was heated to 120 °C, which resulted in partial melting of polyethylene. At the last stage, the obtained sample was cooled to room temperature, which led to crystallization of polyethylene and the formation of a mechanically strong structure. As a result, we obtained bars of layered composite material (graphite foil—polyethylene, Figure 3).

### 2.3. Measurements

**Figure 4:**
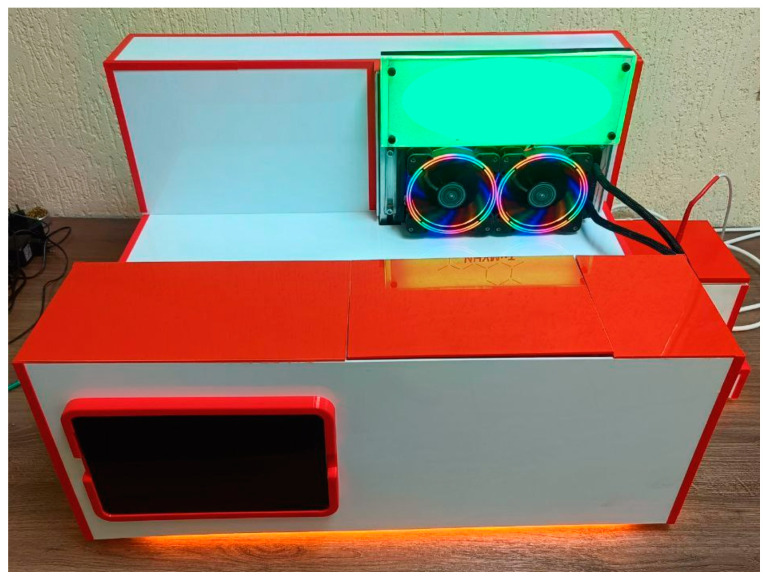
Photograph of the setup for measuring thermal conductivity of functional and structural products.

The in-house-developed setup for measuring the thermal conductivity coefficient implemented the steady-state measurement mode, based on Fourier’s law (Figure 4). Let us recall that the thermal conductivity coefficient, λ [W/m⋅K], characterizes the ability of a material to conduct heat and is calculated on the basis of the steady-state heat flux through a sample and its geometric parameters (Equation (2)):

(2) $$\lambda =\frac{Q\cdot ∆l}{S\cdot \left(T_{1}-T_{2}\right)}$$

where Q is the heat flux through the sample in steady state; ∆l is the distance between the temperature-controlled extremities of the sample; S is the cross-sectional area of the sample; T_1_ is the temperature of the hot edge of the sample; and T_2_ is the temperature of the cold edge of the sample.

#### 2.3.1. The Basic Units of the Developed Device

The developed device for measuring thermal conductivity includes:

(1) Heating unit consisting of a ceramic heating element and resistance temperature detectors to monitor the input heat flux;
(2) Cooling unit consisting of a Peltier element with a water-cooling system and resistance temperature detectors to monitor the output heat flux;
(3) Central unit including a sample compartment and resistance temperature detectors with a special mechanism for moving them.

The heating and cooling units are connected to the test sample using thermal pads, which ensure tight contact and efficient heat transfer. To prevent heat exchange with the environment, the heating unit, cooling unit, and central unit are thermally insulated.

#### 2.3.2. Block Diagram of the Setup Control

The electronic control unit of the automated system for the measurement of the thermal conductivity of functional and structural products consists of a central processor, auxiliary controllers, high-precision analog-to-digital converters (ADS 1220), three-wire resistance temperature detectors (RTD), MOSFET modules, LC filters, load cells, and stepper motor drivers.

**Figure 5:**
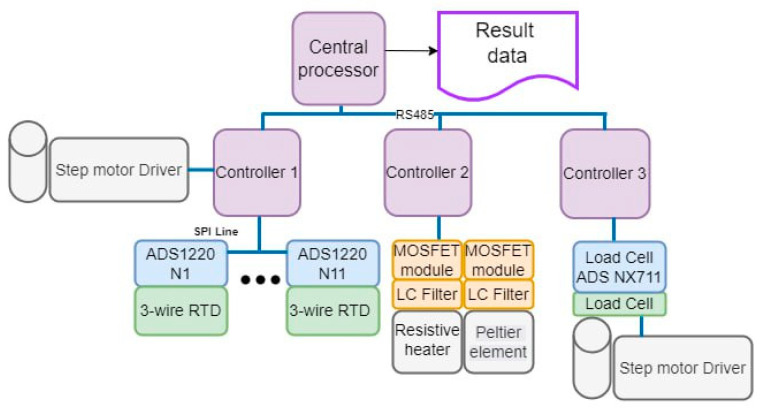
Block diagram of the setup control.

All controllers and the processor are connected by a common data exchange bus. The central processor sequentially interrogates the auxiliary controllers, each of which sends back its data and executes the received commands. For convenience, the auxiliary controllers are divided according to the functional tasks they perform (Figure 5):

- Auxiliary controller 1 is responsible for receiving temperature data on the setup (heating unit, sample, cooling unit), and it also controls the stepper motor which clamps the RTDs;
- Auxiliary controller 2 is responsible for controlling and operating the resistive heater (heating unit) and the Peltier module (cooling unit);
- Auxiliary controller 3 is responsible for clamping the resistive heater after polling the load cell, as well as for controlling its stepper motor.

### 2.4. Modelling Heat Transfer in the Setup

**Figure 6:**
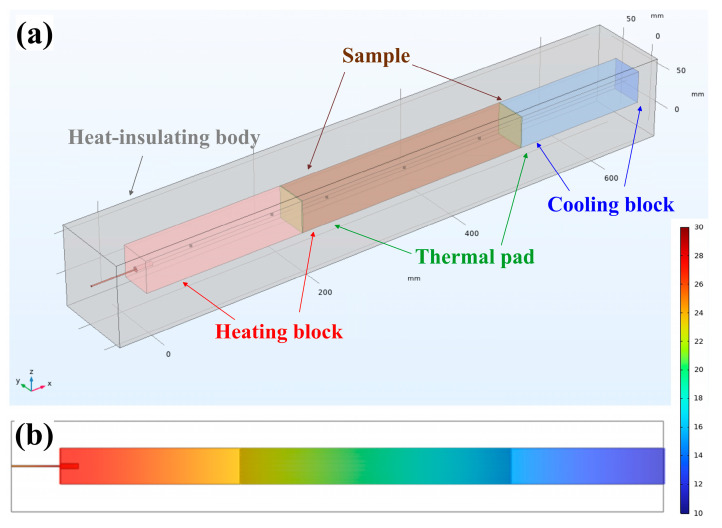
Geometric model of the setup (**a**) and temperature field distribution in the setup (**b**).

Based on the experimental setup, a mathematical model [43,44,45] was developed for simulating heat transfer in flexible graphite foil samples (Figure 6a). The assumptions of the mathematical model [46,47,48] and the finite element method (FEM) [49,50] which were used are described in detail in our previous work [41].

After having modeled the working area of the setup (Figure 6a), one must assign a material to each structural element (aluminum, copper, graphite, polyethylene, etc.). Next, to model the temperature fields, three input parameters must be set for each material: density (ρ), heat capacity ($c_{p}$), and thermal conductivity (λ). Note that the thermal conductivity tensor was set separately for each layer of graphite foil and for each layer of polyethylene (Figure 3d). To compare the modeling data with the experimental data, heat exchange with the environment was taken into account.

The next step is to set the time range and the measurement step. In our case, we recorded temperature change once per minute.

It is also worth noting that, since the dimensions of the layered sample (Figure 3d) are very small compared to the other parts of the setup, it is necessary to be careful in constructing the calculation grid for the setup when modeling using finite element methods.

The agreement of experimental data and FEM data was monitored using output data for the aluminum sample (namely, the process of reaching steady state and distribution of temperature fields along the setup) (Figure 6b) [41].

## 3. Results

### 3.1. Study of Thermal Conductivity of Graphite Samples

**Figure 7:**
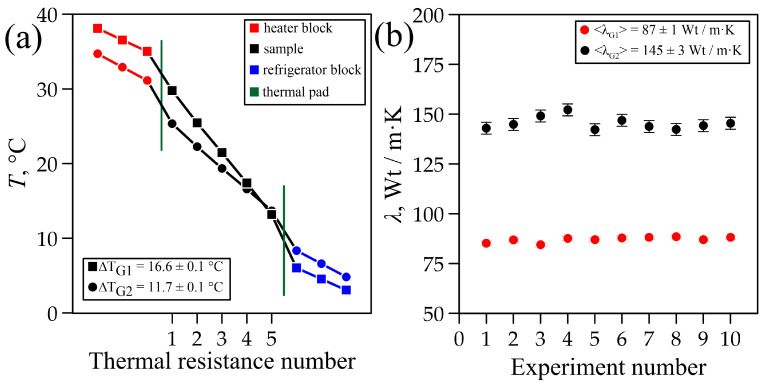
Temperature distribution in the setup (**a**) and thermal conductivity coefficients (**b**) of samples G_1_ и G_2_.

To measure the thermal conductivity coefficient of solid graphite materials (Figure 1), two isotropic samples were made of artificial graphite G_1_ (density 1.8 g/cm^3^) and natural graphite G_2_ (density 1.7 g/cm^3^). The measurement results are shown in Figure 7.

Artificial graphite sample G_1_ (density 1.8 g/cm^3^) was measured in the experimental setup at room temperature (T_room_ = 22 ± 1 °C). The experimental setup reached a steady state within ∆t = 0.5 h, when the heat fluxes in the heater unit (Q_1_ = 11.1 ± 0.7 W) and in the cooling unit (Q_2_ = 10.7 ± 0.5 W) became equal. In a series of 10 measurements with a temperature difference of ∆T = 16.6 ± 0.1 °C (Figure 7a), the thermal conductivity of the artificial graphite sample was <λ_G1_> = 87 ± 1 W/m·K (Figure 7b).

Natural graphite sample G_2_ (density 1.7 г/cm^3^) was measured in the experimental setup at room temperature (T_room_ = 22 ± 1 °C). The experimental setup reached a steady state within ∆t = 0.5 h, when the heat fluxes in the heater unit (Q_1_ = 12.9 ± 0.7 W) and in the cooling unit (Q_2_ = 12.8 ± 0.7 W) became equal. In the series of 10 measurements with a temperature difference of ∆T = 11.7 ± 0.1 °C (Figure 7a), the thermal conductivity of the natural graphite sample was <λ_G2_> = 145 ± 3 W/m·K (Figure 7b).

### 3.2. Study of Thermal Conductivity of Flexible Graphite Foil (GF)

Flexible graphite foil (GF) is a promising innovative material with high thermal conductivity. Its thermal conductivity coefficient varies between 5 and more than 500 W/m·K; thus, considering a density of 1.0 g/cm^3^, λ_GF_ is comparable to the thermal conductivity of aluminum. It is also possible to increase it by modifying the technology of GF production, which makes it possible to expand the application range of products based on graphite foils.

**Figure 8:**
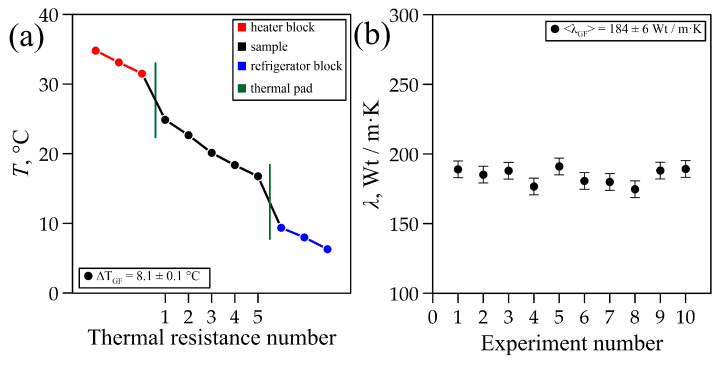
Temperature distribution in the setup (**a**) and thermal conductivity coefficient of the GF sample (**b**).

The graphite foil sample, manufactured using the “in-plane” technology, was measured in the experimental setup at room temperature (T_room_ = 22 ± 1 °C). The experimental setup reached a steady state within a time of ∆t = 0.5 h, with almost equal heat fluxes in the heating unit (Q_1_ = 11.9 ± 1.9 W) and in the cooling unit (Q_2_ = 11.1 ± 0.6 W). In a series of 10 measurements at a temperature difference of ∆T = 8.1 ± 0.1 °C (Figure 8a), the thermal conductivity of the graphite foil sample was <λ> = 184 ± 6 W/m·K (Figure 8b).

### 3.3. X-Ray Studies of Graphite Materials

Properties of the composite product were determined by the parameters of its components: graphite foil and polyethylene. We studied the thermal conductive properties of the GF sample along its rolling direction; the GF thermal conductivity coefficient was 450 times higher than that of polyethylene. Therefore, the thermal conductivity of the composite product was determined by the characteristics of the graphite foil.

**Figure 9:**
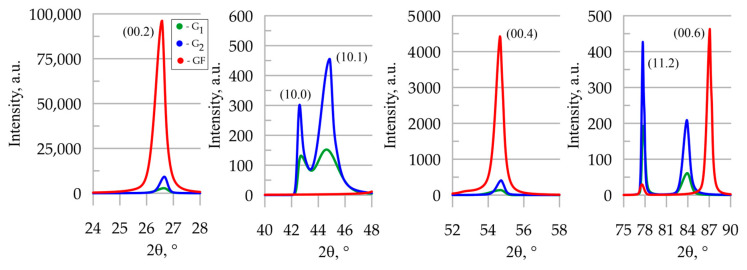
X-ray diffraction spectra of samples.

The analysis of XRD patterns (Figure 9) of graphite foil with a density of 1.0 g/cm^3^ (which is the basic component of the studied composite) revealed a pronounced preferred orientation of basal planes in the rolling plane of the foil. This was evident from the presence of intense reflection (0002) and the absence of reflections (10-10) and (10-11) from prismatic and pyramidal planes, indicating a pronounced anisotropy of physical properties.

The opposite situation was observed in high-density graphite samples G_1_ и G_2_ ($\rho_{G1}$ = 1.8 g/cm^3^, $\rho_{G2}$ = 1.7 g/cm^3^); these were highly isotropic, judging from the presence of high-intensity reflections from the (10-10) and (10-11) planes, which are different from the basal planes. To assess the degree of isotropy of the graphite materials’ samples, we used ratios of integral X-ray reflection intensities of prismatic or pyramidal planes to those of basal planes, as given in Table 1.

Such ratios allow for an intercomparison of the characteristics of the samples: the greater the parameter values, the lower the anisotropy, i.e., the more uniform the directional distribution of magnitudes of properties in the sample is. The obtained results indicate that sample G_1_ was more isotropic than all the others. This was because it was prepared from artificial graphite, which is usually characterized by an isotropic distribution of particle size and nano-crystallites. The analysis of XRD patterns reveals various reflections of sample G_1_ with considerable peak widths at half height (B), which is indicative of a finer graphite substructure. This is confirmed by the calculation of the sizes of graphite nano-crystallites using the Scherrer method [51,52]. High isotropy and particle fineness, associated with an elevated number of inter-crystallite boundaries, contribute to a decrease in thermal conductivity in such a material, which was confirmed by experimental data (Figure 7b and Figure 8b).

The structural features of samples G_1_ and G_2_ were basically similar, but still have some differences. Sample G_2_ was less anisotropic compared to the graphite foil sample, but more anisotropic than artificial graphite G_1_. Its B values were lower than those of samples G_1_ and GF, which was due to an increased size of nano-crystallites in the sample and, consequently, less important micro-deformations. This factor, together with higher anisotropy (as compared to G_1_) and fewer defects, gave rise to the increased thermal conductivity of such a material (Figure 7b).

The thermal conductivity of the composite product was determined by the characteristics of the graphite foil. Due to the high degree of orientation of the basal planes in the rolling plane, and despite the fact that the crystallite size in GF was smaller and the microdistortions were higher than in G_2_, the GF composite product had the highest thermal conductivity (Figure 8b) among the graphite materials in question. It is also worth noting that the composite material consisted of low-density graphite foil (1.0 g/cm^3^). Technologically, it is possible to create denser graphite foil, so a composite material based on higher thermal conductivity values can be achieved, competing with products of the same shape made of aluminum and copper.

### 3.4. Modelling of Temperature Fields in Graphite Foil

Temperature fields were calculated with the help of computer modeling based on the finite element method (FEM). We performed simplified modeling of the working area of the developed setup, which included: a heating unit, a cooling unit, thermal pads, a test sample, and heat-insulating housing (Figure 6b).

Based on the experimental data on the GF sample obtained by the “in-plane” method, the inverse problem was solved and the following characteristics were obtained (they were used as initial conditions in computer modeling): the power of the heating and the cooling units was Q = 8.0 W, and the tensor of the thermal conductivity coefficient for the highly anisotropic GF material, λ_GF_, was $\left(\begin{array}{ccc} 184 & 0 & 0\\ 0 & 184 & 0\\ 0 & 0 & 7 \end{array}\right)$. The thermal conductivity in the *z*-direction, λ_z_, was supposed to be 7 W/m·K based on previous experimental findings [19,20].

The discrepancy between the simulation and experimental data is insignificant. The main contribution to the difference between the simulation and experimental results was the presence of thermal resistance in the sample when measuring the thermal conductivity coefficient. Detailed results on the perturbations of temperature fields in the presence or absence of thermal resistances are given in [41].

**Figure 10:**
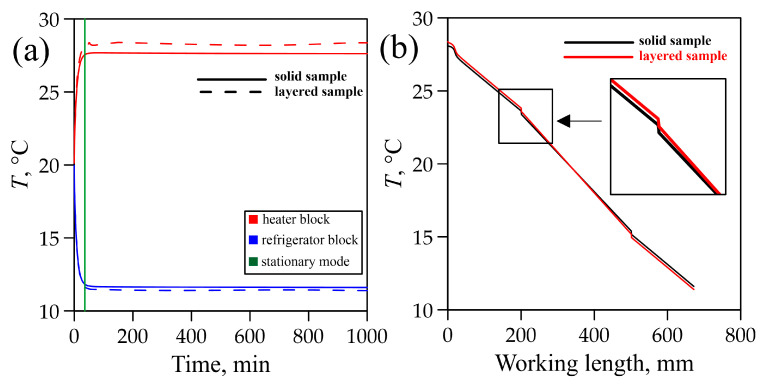
Results of modeling the solid graphite foil sample: time to steady state (**a**) and temperature distribution along the setup (**b**).

At the first stage of computer modeling, the graphite foil sample was represented as a solid bar, anisotropic in the *z*-direction (Section 3.1). Analogously to the experimental data, we modeled temperature distribution along the working area of the setup and the time to reach a steady state (Figure 10a,b). We assumed the time to reach a steady state to be the time after which the temperature change was ≤0.01 °C.

At the second stage of computer modeling, the sample was represented by a composite material: graphite foil (GF) + polyethylene (P). The thickness of the GF layer was 0.38 mm, and the thickness of the P layer was 0.02 mm. The number of layers in such a model composite material is k = 100. The thermal conductivity tensor of P, λ_P_, was $\left(\begin{array}{ccc} 0.40 & 0 & 0\\ 0 & 0.40 & 0\\ 0 & 0 & 0.35 \end{array}\right)$ [53,54].

Numerical modeling yielded the following results:

(1) The time to reach a steady state was the same in both models and was equal to 36 min;
(2) The behavior of the curves describing the temperature distribution in the setup also coincided for the two cases (the difference was about 1%) (Figure 10b).

Based on the results obtained from modeling temperature fields in graphite foil samples, it is possible to conclude that solid products made of compacted thermally exfoliated graphite (TEG) and products made of composite (graphite foil + polyethylene) material can be used interchangeably due to their coinciding thermal conductive properties. The manufacturing of large-sized solid products from TEG presents technological difficulties, in contrast to the composite product.

## 4. Conclusions

Using the developed equipment for measuring the thermal conductivity of functional and structural products by the steady-state Fourier’s law-based method, experimental values for high-purity aluminum (99.999 wt.% Al) and copper (99.990 wt.% Cu) were obtained: <λ_Al_> = 243 ± 3 W/m·K and <λ_Cu_> = 405 ± 4 W/m·K at a room temperature of T = 22 ± 1 °C. These results are consistent with reported literature values, demonstrating the stable operation of the equipment using materials with high thermal conductivity.

The thermal properties of carbon-based materials, including artificial graphite ($\rho_{G1}$ = 1.8 g/cm^3^) and natural graphite ($\rho_{G2}$ = 1.7 g/cm^3^), were studied. Thermal conductivity coefficients were <λ_G1_> = 87 ± 1 W/m·K and <λ_G2_> = 145 ± 3 W/m·K, respectively. Additionally, the thermal conductivity of a composite material based on flexible graphite foil was measured as <λ_GF_> = 184 ± 6 W/m·K at a room temperature of T = 22 ± 1 °C.

The structural features of the studied graphite materials depend on their nature. Artificial graphite, with its isotropic structure resulting from its production method, has the lowest thermal conductivity. It is shown that, in addition to low anisotropy, the fine particle size and the presence of defects in graphite contribute to the deterioration of thermal properties. Natural graphite shows higher values of thermal conductivity than artificial graphite, while remaining fairly isotropic. It is shown that the thermal conductivity coefficient is affected by the size of graphite crystallites. We have also found that a composite made of graphite foil has higher thermal conductivity in its rolling plane than the materials mentioned above.

Measuring thermal conductivity of thin graphite foil samples is a difficult metrological task, so we proposed to prepare a solid rectangular sample using alternating layers of thin graphite foil connected by layers of ultra-thin polyethylene films. Computer simulation revealed that both solid products from compacted thermally exfoliated graphite and products from the composite material made of 100 layers of thin graphite foil and 99 layers of polyethylene reached steady-state mode in in approximately 36 min. The temperature distribution curves from the calculation and from the experiment showed a deviation of less than 1% between them. This result substantiates our proposed approach to measuring the thermal conductivity of flexible graphite foil by preparing a multilayer composite material.

Analysis of the results indicates the great potential of flexible graphite foil and composites based on it as heat-conducting interfaces for cooling systems. Given their lower weight compared to aluminum and copper, such a material is promising for use in the aircraft and space industries, in microelectronics, and in lithium-ion batteries.

Further studies in this direction will focus on extending the working range of the experimental setup to include temperatures between −50 and 250 °C. Efforts on studying the thermal properties of high-density flexible graphite foils at different temperatures are also planned.

## Figures and Tables

**Table 1:** Structural parameters of the studied materials.

Sample	$\frac{I_{\left(10\bar{1}1\right)}}{I_{\left(0002\right)}}$	$\frac{I_{\left(10\bar{1}0\right)}}{I_{\left(0002\right)}}$	$\frac{I_{\left(11\bar{2}0\right)}}{I_{\left(0004\right)}}$	$B_{0002}$, deg.	$B_{10\bar{1}0}$, deg.	$B_{10\bar{1}1}$, deg.	$B_{11\bar{2}0}$, deg.	*a*, pm	*c*, pm	*L_c_*, pm
G_1_	0.175	0.066	0.944	0.491	0.780	1.860	0.410	245.2 ± 0.5	669.8 ± 1.5	13 ± 4
G_2_	0.156	0.057	0.790	0.321	0.420	1.020	0.294	245.4 ± 0.4	669.4 ± 1.5	23 ± 6
GF	-	-	0.058	0.396	-	-	0.550	246.3 ± 1.9	670.8 ± 0.3	19 ± 2

## Data Availability

The original contributions presented in this study are included in the article. Further inquiries can be directed to the corresponding author.
